# Effect of flatfoot correction on the ankle joint following lateral column lengthening: A radiographic evaluation

**DOI:** 10.1371/journal.pone.0286013

**Published:** 2023-11-02

**Authors:** Ji Hye Choi, Yoon Hyo Choi, Dae Hyun Kim, Dong Yeon Lee, Seungbum Koo, Kyoung Min Lee

**Affiliations:** 1 Department of Orthopedic Surgery, Seoul National University Bundang Hospital, Seongnam, South Korea; 2 Department of Orthopedic Surgery, Seoul National University Hospital, Seoul, South Korea; 3 Department of Mechanical Engineering, Korea Advanced Institute for Science and Technology, Daejon, South Korea; Università degli Studi di Milano: Universita degli Studi di Milano, ITALY

## Abstract

**Objectives:**

The effects of foot deformities and corrections on the ankle joint without osteoarthritis has received little attention. This study aimed to investigate the effect of flatfoot correction on the ankle joint of patients without osteoarthritis.

**Methods:**

Thirty-five patients (24 men and 11 women; mean age 17.5 years) who underwent lateral column lengthening for flatfoot deformities were included. The mean postoperative follow-up period was 20.5 months (standard deviation [SD]: 15.7 months). Radiographic indices were measured pre- and postoperatively, including anteroposterior (AP) and lateral talo-first metatarsal angles, naviculocuboid overlap, position of the articulating talar surface, and lateral talar center migration. Postoperative changes in the radiographic indices were statistically analyzed.

**Results:**

There was significant postoperative improvement in flatfoot deformity in terms of AP and lateral talo-first metatarsal angles (p<0.001 and p<0.001, respectively) and naviculocuboid overlap (p<0.001). On lateral radiographs, the talar articulating surface dorsiflexed by 7.3% (p<0.001), and the center of the talar body shifted anteriorly by 0.85 mm (p<0.001) postoperatively.

**Conclusions:**

Flatfoot correction using lateral column and Achilles tendon lengthening caused dorsiflexion and an anterior shift of the articular talar body in patients without osteoarthritis. Correction of flatfoot deformity might affect the articular contact area at the ankle joint. The biomechanical effects of this change need to be investigated further.

## Introduction

The talus is a key structure modulating ankle joint motion and the structure of the foot, which accepts and dissipates the ground reaction force [1]. Therefore, foot deformity can affect the structure and function of the ankle joint via the talus [2,3]. Although foot deformities concomitant with ankle osteoarthritis (OA) are drawing the attention of surgeons when planning and determining the extent of surgical treatment for ankle OA [4], the effects of foot deformity or deformity correction on the ankle joint without OA are not being considered.

The talus is plantar flexed and internally rotated within the ankle mortise in patients with flatfoot deformities [5]. However, despite this preexisting knowledge, the fact that the talus is rotationally subluxated to plantar flexion within the ankle joint has not been studied, resulting in scarce evidence on this issue.

A recent study raised interest in the role of flatfoot deformity in the development and progression of ankle OA and reported radiographic and clinical improvement following flatfoot correction by subtalar arthrodesis for posterior ankle OA [6]. This study highlights that flatfoot deformity could be directly associated with specific types of ankle OA. However, the effect of flatfoot correction on the ankle joint without OA has not yet been demonstrated, and this issue is important because correcting flatfoot deformities could be a potential preventive measure for posterior ankle OA.

Therefore, we hypothesized that flatfoot correction would affect the ankle joint in patients without OA. The aim of this study was to evaluate radiographic changes in the foot and ankle following lateral column lengthening in young and adolescent patients without OA.

## Materials and methods

This retrospective study was approved by the institutional review board of our institution (a tertiary referral center for orthopedic surgery), and the requirement for informed consent from the participants was waived due to the retrospective nature of the study.

### Subjects

**Fig 1:**
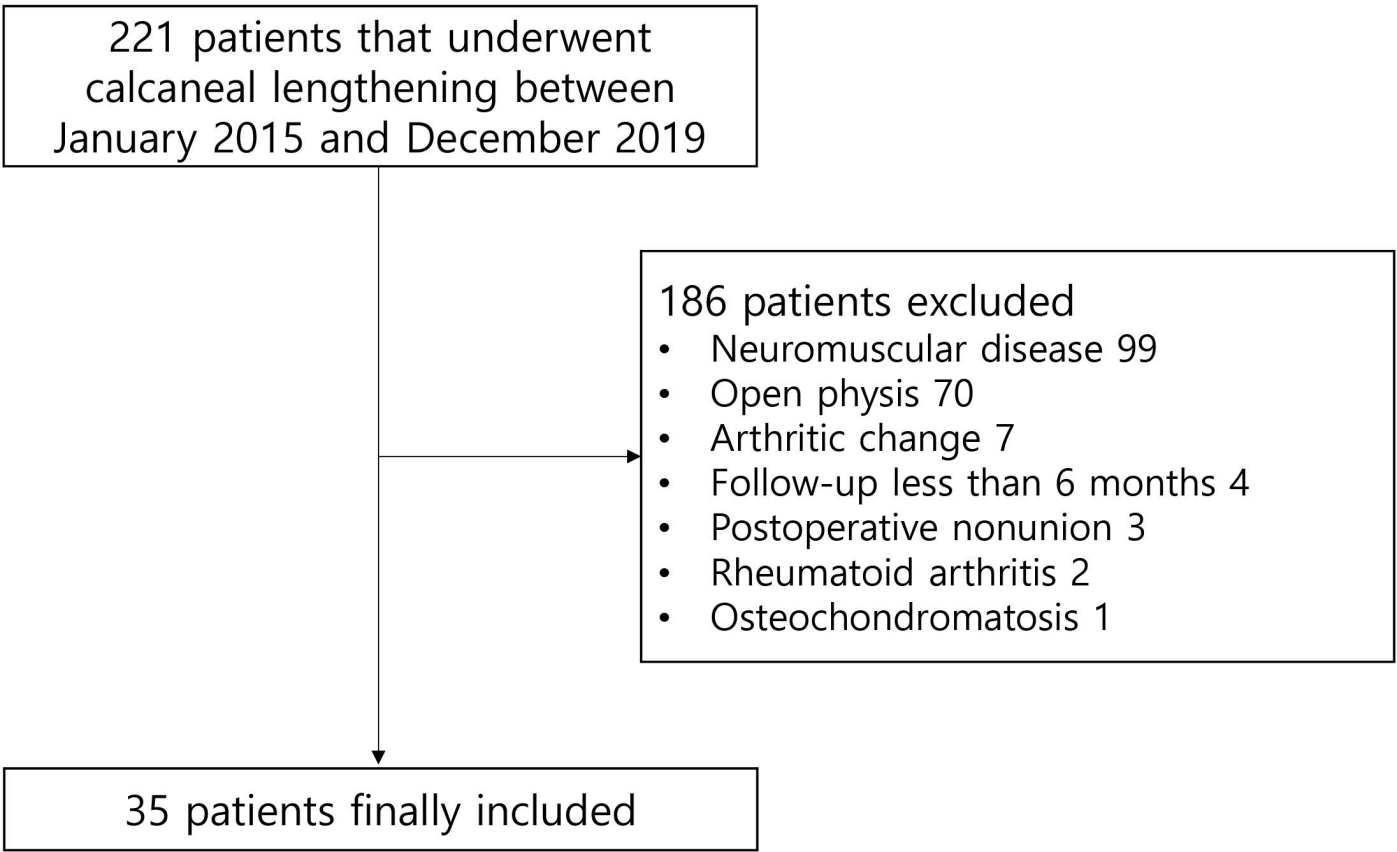
Flow diagram showing patient selection.

Consecutive patients who underwent lateral column lengthening procedures for flatfoot deformity between January 2015 and December 2019 at our hospital were enrolled from electronic medical records. The exclusion criteria were as follows: 1) congenital anomaly; 2) neuromuscular diseases; 3) skeletally immature patients with open physis on foot and ankle radiographs; 4) previous surgery, trauma, tumor, or infection that could change the normal anatomy; 5) degenerative changes of the foot and ankle; 6) rheumatoid or inflammatory arthritis; 7) inadequately taken X-rays; 8) postoperative nonunion of bone graft; and 9) postoperative follow-up of less than 6 months (Fig 1). Demographic data of the patients were collected, including age, sex, and body mass index (BMI). The age- and sex-matched control group was recruited from the contralateral uninjured foot of the patients who visited the hospital for a foot or ankle sprain and underwent weight-bearing radiography.

A total of 35 patients and 35 controls were included in this analysis. The mean ages of the patients and control group were 17.5 years (SD 4.5 years) and 18.1 years (SD 3.5 years) (p = 0.558). There were 11 men and 24 women in the patient group and 14 men and 21 women in the control group (P = 0.618). BMI was not significantly different between the two groups (p = 0.569) (Table 1).

**Table 1:** Data summary.

	Preoperative Patients	Control	p-value
No. of patients	35	35	-
Age (years)	17.5 (SD 4.5)	18.1 (SD 3.5)	0.558
Male: Female	11: 24	14: 21	0.618
Height (cm)	167.5 (SD 8.6)	167.3 (SD 8.9)	0.948
Weight (kg)	67.3 (SD 17.9)	61.9 (SD 19.7)	0.346
BMI (kg/m^2^)	23.8 (SD 4.6)	23.0 (SD 4.0)	0.569
Side (right: left)	14: 21	19: 16	0.338
Radiographic measurements			
AP talo-1MT (°)	20.3 (SD 6.3)	7.3 (SD 4.0)	<0.001
Lat talo-1MT (°)	28.7 (SD 8.1)	7.3 (SD 4.1)	<0.001
Naviculocuboid overlap	0.74 (SD 0.10)	0.58 (SD 0.13)	<0.001
Position of the articulating surface (%)	56.9 (SD 5.3)	49.5 (SD 4.5)	<0.001
Lateral talar center migration (mm)	-0.31 (SD 0.46)	0.07 (SD 0.42)	0.001

BMI, body mass index, AP talo-1MT, anteroposterior talo-first metatarsal angle; Lat talo-1MT, lateral talo-first metatarsal angle.

### Radiographic examination

Radiographs of the feet were taken using a DigitalDiagnost X-ray machine (Philips Healthcare, Amsterdam, The Netherlands), according to our protocol. The anteroposterior (AP) weight-bearing view was obtained with the central beam angled at 15° to the vertical axis and centered between the feet at the level of the midtarsal joint with the patient standing barefoot. The feet were 10 cm apart and their medial borders were parallel. Lateral weight-bearing foot and ankle radiographs were taken separately for each foot standing with the beam focusing on the medial cuneiform. The radiographic settings were 60 kVp and 10 mA at a source-to-image distance of 110 cm. All radiographic images were digitally acquired using a picture archiving and communication system (PACS; INFINITT Healthcare Co., Ltd., Seoul, South Korea), and radiographic measurements were performed using the PACS software.

### Radiographic measurements and interobserver reliability

A consensus-building session was held between three orthopedic surgeons with 25, 21, and 6 years of orthopedic experience. The following radiographic measurements were selected and evaluated based on previous studies [6–8]: AP talo-first metatarsal angle (AP talo-1MT) [7,8], lateral talo-first metatarsal angle (Lat talo-1MT) [7,8], naviculocuboid (NC) overlap [7,8], and anterior talar center migration (ATCM) [6]. In addition, the position of the articulating surface of the talar body was quantitatively measured to evaluate the postoperative changes in talar dorsiflexion.

**Fig 2:**
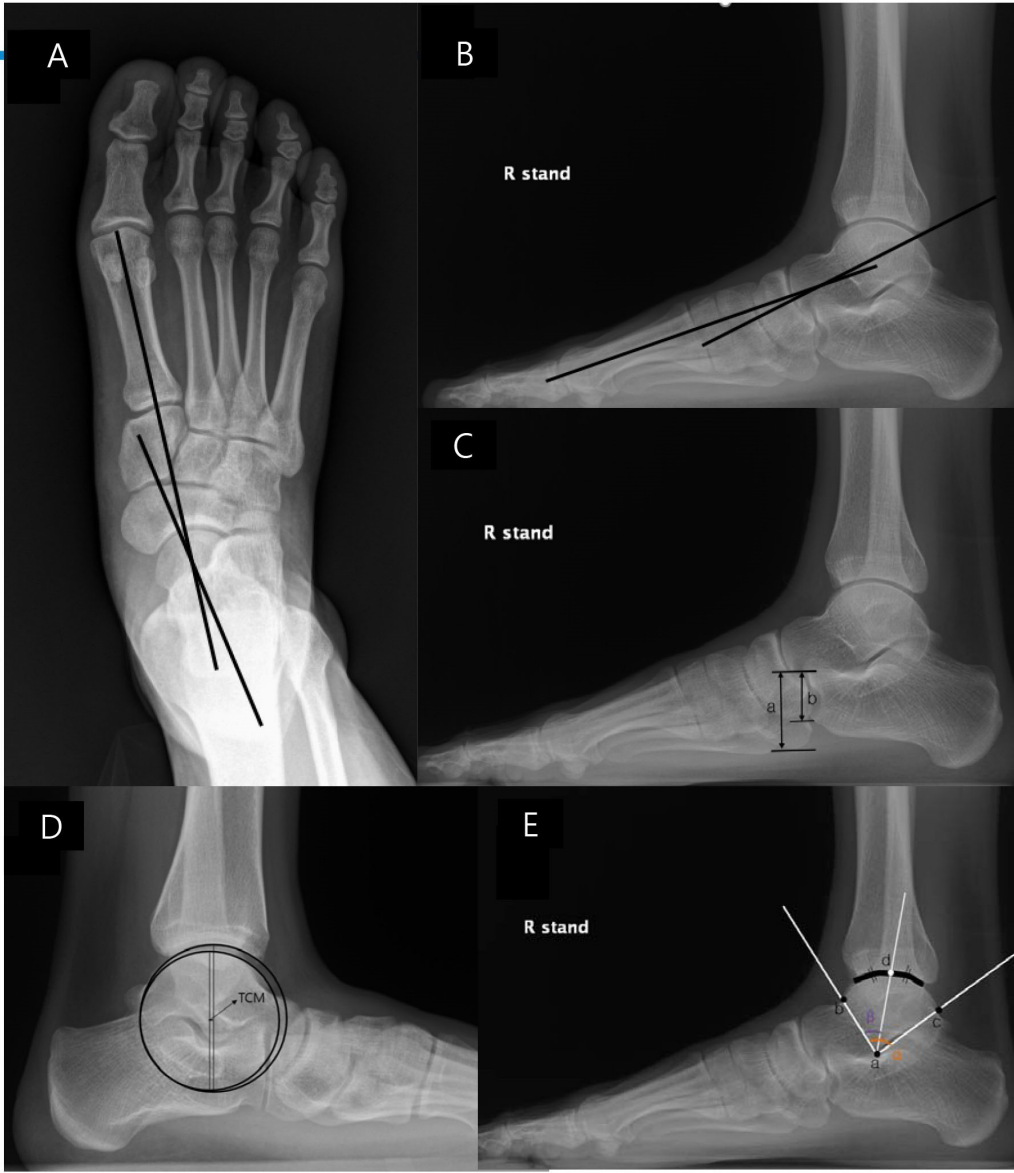
Radiographic measurements. A: AP talo-1MT is measured between the longitudinal axis of talus and that of the first metatarsal on the AP foot X ray. B: Lat talo-1MT is the angle between the longitudinal axis of the talus and that of the first metatarsal on the lateral foot X ray. C: NC overlap (b/a) is the vertical height of overlap between the navicular and cuboid (b) divided by that of the cuboid (a). D: ATCM is the anterior displacement of the center of a circle fitted to the tibial articular surface to that of another circle fitted to the talar articular surface. E: Position of the articulating surface is the angular position of the bisecting point of the contact surface between the tibia and talus within the total arch of the talar articulating surface (β/α; a: tip of the talar lateral process, b: anterior tip of the talar articular surface, c: posterior tip of the talar articular surface, d: bisecting point of the contact surface between the tibia and talus).Two orthopedic surgeons, with 6 and 3 years of orthopedic experience, participated in the reliability testing. After a consensus-building session for radiographic measurements, each surgeon performed radiographic measurements without any clinical information for a predetermined number of radiographic images that were presented in random order by a research assistant who was not involved in this study. One of the surgeons measured the radiographic parameters for all patients pre-and postoperatively, following interobserver reliability testing.

On weight-bearing AP foot radiographs, AP talo-1MT was measured between the longitudinal axis of the talus and the first metatarsal (Fig 2A). On weight-bearing lateral foot and ankle radiographs, the Lat talo-1MT was the angle between the longitudinal axes of the talus and the first metatarsal (Fig 2B). NC overlap was defined as the overlapped portion of the navicular and cuboid divided by the vertical height of the cuboid (Fig 2C). ATCM was defined as the distance between a vertical line passing through the center of a circle fitted to the distal tibial articular surface and another vertical line passing through the center of a circle fitted to the talar articular surface (Fig 2D). The position of the articulating surface of the talar body was the bisecting point of the contact surface between the tibia and talus within the anterior and posterior points of the talar articular surface (Fig 2E).

### Surgical technique

Calcaneal lengthening with a minor modification of the Evans technique was performed by the senior author (K.M.L., fellowship-trained foot ankle surgeon with > 10 years of experience) in accordance with previously published protocols [9]. Transverse osteotomy was performed between the anterior and middle facets of the calcaneus. Gradual distraction of the gap between proximal and distal calcaneal fragments by a laminar spreader was done until the intraoperative correction of the heel valgus was satisfied. A trapezoidal-shaped iliac crest allograft was inserted in the distracted area. Although a typical operation involves around 1 cm of lengthening, the exact amount differs individually, depending on the patient’s foot size and the severity of the heel valgus deformity. Subsequently, a Z-plasty was performed for peroneus brevis and Achilles tendon lengthening. The lengthened Achilles tendon was repaired with 5 degrees of ankle plantar flexion with the knee joint extended.

### Statistical analysis

Descriptive statistical analysis was conducted, including the mean and standard deviation (SD) for continuous variables and proportions for categorical variables. Data normality was determined using the Kolmogorov-Smirnov test. Comparisons of means between pre- and postoperative variables were performed using a paired t-test. The chi-square test was used to compare proportions. Correlations between continuous variables were analyzed using Pearson’s correlation coefficients.

Interobserver reliability was tested using the intraclass correlation coefficient (ICC) with a two-way random effects model, assuming a single measurement and absolute agreement. The sample size for the reliability test was calculated with an ICC target value of 0.9 and 95% confidence interval (CI) width of 0.2. The minimum number of interobserver reliabilities for the two raters was 15, based on Bonnett’s approximation [10]. All statistical analyses were performed using the SPSS version 20.0 (IBM Corp., Armonk, NY, USA), and statistical significance was accepted when p-values were <0.05.

## Results

The mean follow-up period was 20.6 months (SD, 15.7) in the patient group and the inter-observer reliabilities of the radiographic measurements were satisfactory (Table 2).

**Table 2:** Interobserver reliabilities of radiographic measurements.

	ICC	95% CI	p-value
AP talo-1MT (°)	0.877	0.633–0.959	<0.001
Lat talo-1MT (°)	0.917	0.753–0.972	<0.001
Naviculocuboid overlap	0.813	0.443–0.937	0.002
Position of the articulating surface (%)	0.807	0.425–0.953	0.002
Lateral talar center migration (mm)	0.859	0.581–0.953	<0.001

AP talo-1MT, anteroposterior talo-first metatarsal angle; Lat talo-1MT, lateral talo-first metatarsal angle.

Radiographic measurements showed significant postoperative improvement. The position of the articulating surface of the talar body, which was the dorsiflexion of the articulating surface of the talar body, significantly changed postoperatively (p<0.001). The talar body center showed significant anterior migration postoperatively (p<0.001) (Table 3).

**Table 3:** Postoperative radiographic changes following lateral column lengthening.

	Preoperative measurements	Postoperative measurements	p-value
AP talo-1MT (°)	20.3 (SD 6.3)	4.0 (SD 4.8)	<0.001
Lat talo-1MT (°)	28.7 (SD 8.1)	9.9 (SD 5.2)	<0.001
Naviculocuboid overlap	0.74 (SD 0.10)	0.55 (SD 0.10)	<0.001
Position of the articulating surface (%)	56.9 (SD 5.3)	49.6 (SD 4.1)	<0.001
Lateral talar center migration (mm)	-0.31 (SD 0.46)	0.55 (SD 0.47)	<0.001

AP talo-1MT, anteroposterior talo-first metatarsal angle; Lat talo-1MT, lateral talo-first metatarsal angle.

NC overlap (p = 0.357) and position of the articulating surface (p = 0.964) did not show significant differences between the control group and postoperative patients, while AP talo-1MT, Lat talo-1MT, and lateral talar center migration were significantly different (Table 4).

**Table 4:** Comparison of radiographic measurements between postoperative patients and the control group.

	Postoperative measurements	Control group	p-value
AP talo-1MT (°)	4.0 (SD 4.8)	7.3 (SD 4.0)	0.003
Lat talo-1MT (°)	9.9 (SD 5.2)	7.3 (SD 4.1)	0.023
Naviculocuboid overlap	0.55 (SD 0.10)	0.58 (SD 0.13)	0.357
Position of the articulating surface (%)	49.6 (SD 4.1)	49.5 (SD 4.5)	0.964
Lateral talar center migration (mm)	0.55 (SD 0.47)	0.07 (SD 0.42)	<0.001

AP talo-1MT, anteroposterior talo-first metatarsal angle; Lat talo-1MT, lateral talo-first metatarsal angle.

Postoperative changes in AP talo-1MT were significantly correlated with those in Lat talo-1MT (r = 0.459, p = 0.005) and NC overlap (r = 0.509, p = 0.002) (Table 5).

**Table 5:** Correlation among the radiographic changes.

	BMI	Follow-up	ΔAP talo-1MT	ΔLat talo-1MT	ΔNCoverlap	ΔPosition of the articulating surface	ΔATCM
Age	0.154 p = 0.159	-0.187 p = 0.122	-0.349 p = 0.040	-0.096 p = 0.583	-0.032 p = 0.855	-0.087 p = 0.619	-0.073 p = 0.676
BMI		-0.126 p = 0.299	0.111 p = 0.525	0.186 p = 0.284	-0.065 p = 0.710	-0.043 p = 0.805	0.372 p = 0.028
Follow-up			0.224 p = 0.196	0.249 p = 0.149	0.045 p = 0.797	0.216 p = 0.213	0.198 p = 0.253
ΔAP talo-1MT				0.459 p = 0.005	0.509 p = 0.002	0.086 p = 0.622	0.232 p = 0.180
ΔLat talo-1MT					0.384 p = 0.023	0.059 p = 0.736	0.110 p = 0.529
ΔNCoverlap						-0.213 p = 0.219	-0.127 p = 0.468
ΔPosition of the articulating surface							0.054 p = 0.756

Δ, postoperative change; BMI, body mass index; AP talo-1MT, anteroposterior talo-first metatarsal angle; Lat talo-1MT, lateral talo-first metatarsal angle; NCoverlap, naviculocuboid overlap.

## Discussion

Flatfoot deformity is a very common orthopedic condition that requires surgical treatment, and many studies have reported satisfactory radiographic and clinical outcomes [10–14]. However, the effect of flatfoot correction on the ankle joint has not been sufficiently investigated. This study highlighted radiographic changes in the ankle joint following lateral column and Achilles tendon lengthening for flatfoot deformity in patients without ankle OA.

Lateral column lengthening effectively corrected the flatfoot deformity. Forefoot abduction (AP talo-1MT), medial foot arch collapse (Lat talo-1MT), and midfoot pronation (NC overlap) significantly improved postoperatively, which is consistent with previous studies [10,11]. The effect of flatfoot correction on the ankle joint has drawn little attention despite it being able to change the talus position. A recent study reported clinical and radiographic improvement in posterior ankle OA concomitant with flatfoot deformity following flatfoot correction by subtalar arthrodesis [6], highlighting the role of flatfoot deformity in the development of ankle OA.

Our study showed that the talar articulating surface with tibial plafond in the flatfoot was significantly posteriorly located due to plantar flexion of the talus compared with that in normal controls. This condition could be considered as plantar flexion rotatory subluxation of the talus. Anatomically, the articular surface of the talar body is narrower on the posterior side than on the anterior side [15], and plantar flexion of the talus narrowed the articular contact surface, which could potentially increase the joint contact stress, leading to the development of OA [16].

Considering previous human in vivo MRI studies [17,18], which have demonstrated the varying distribution of cartilage thickness in the talar dome, we hypothesize the eccentricity of cartilage thickness in patients with planovalgus deformity. Such patients may have thinner posterior articular cartilage, presented as radiographical narrowing of the posterior joint space, but this has not been validated in previous studies.

However, this issue needs to be validated through biomechanical and cadaver studies, although this is a reasonable assumption. Furthermore, if flatfoot deformity is a risk factor for ankle OA, the surgical indication for flatfoot correction to prevent ankle OA may need to be established in the future, in addition to the current surgical indications for relief of flatfoot symptoms.

**Fig 3:**
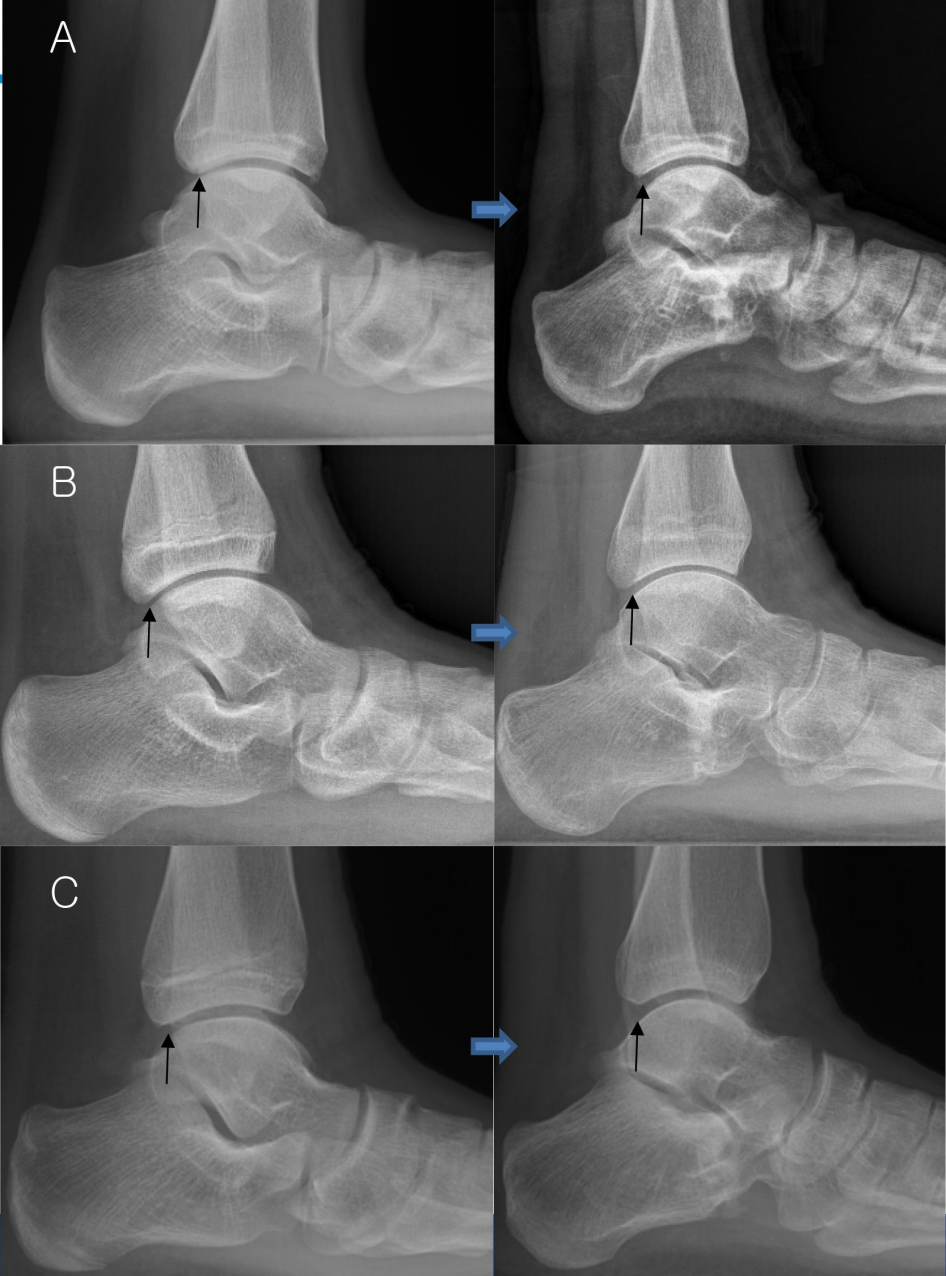
Postoperative radiographic change following calcaneal lengthening. A: A narrow posterior ankle joint interval (arrows) shows improvement at 2 years and 6 months postoperatively in a 20-year-old man. The talus has been dorsiflexed and anteriorly displaced postoperatively. B: A similar postoperative radiographic change in a 14-year-old boy at 11 months postoperatively. C: Another example in a 14-year-old girl at 1 year and 8 months postoperatively.

**Fig 4:**
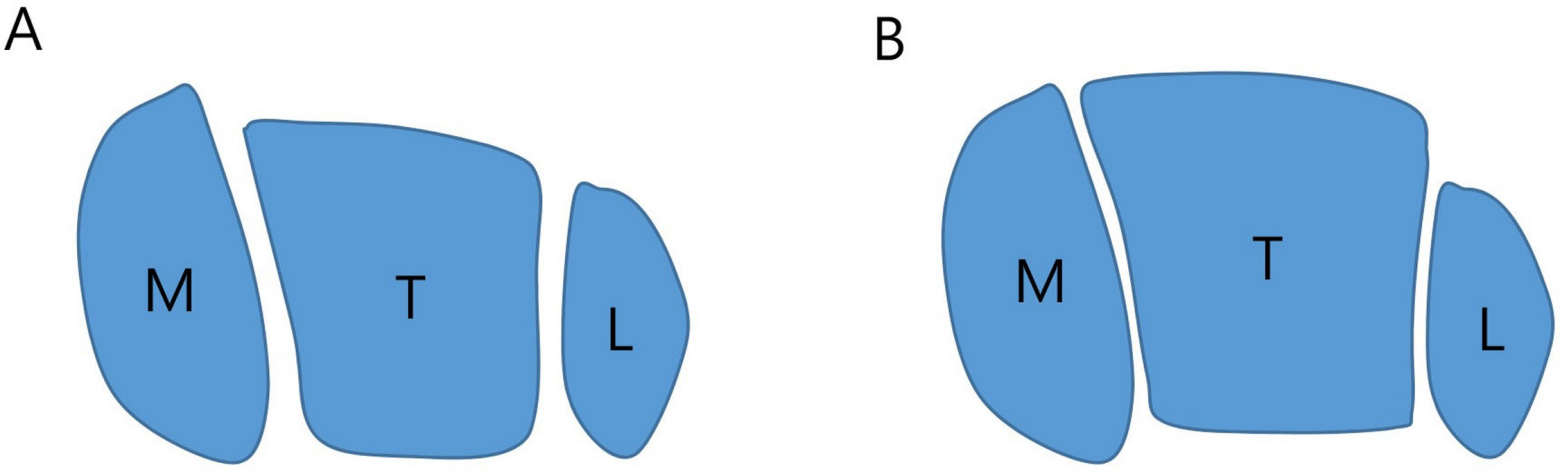
Schematic drawing of the ankle joint in the axial plane (M, medial malleolus; T, talus; L, lateral malleolus). A: With a preoperative flatfoot deformity, the talus is plantar flexed in the ankle joint and a narrower posterior talar dome comes in contact with the tibial plafond. The narrower posterior talar body barely comes in contact with the medial and lateral malleoli on the sides. In turn, the posterior aspect of the ankle joint may receive focused contact and shear force. B: Following flatfoot correction, the talus is dorsiflexed and a wider talar body comes in contact with the tibial plafond and the medial and lateral malleoli. Consequently, the wider contact surface would distribute the load evenly across the ankle joint.

Patients with flatfoot deformity showed a more posteriorly located talar body compared with normal controls, and flatfoot correction by lateral column lengthening caused anterior displacement of the talar body postoperatively (Fig 3). In flatfoot deformity, the plantar-flexed and posteriorly-located talus articulates with the distal tibia through a relatively limited contact surface in the sagittal plane. In turn, the posterior aspect of the ankle joint may receive focused contact and shear force, owing to the anterior inclination of the distal tibial articular surface. However, when the talus dorsiflexes due to the combined effect of Achilles tendon lengthening and lateral column lengthening, the joint contact surfaces between the talar dome and distal tibial plafond increase, but so do the contact surfaces at the medial and lateral gutters. This wider joint contact surface would provide even load distribution across the ankle joint. Additionally, in the axial plane, a wider anterior talar body (dorsiflexed talus following flatfoot correction) is more difficult for posterior displacement within the ankle mortise composed of the tibia and fibula, which, in turn, causes anterior displacement of the talus postoperatively (Fig 4). The rigidity of the width between the tibia and fibula was determined by the flexibility of the tibiofibular syndesmosis, which is considered to be one of the reasons why the amount of anterior displacement of the talus was not correlated with that of flatfoot correction. This issue should be addressed in future studies for an established explanation.

There are some limitations to consider when interpreting the study results. First, the sample size was small, and the postoperative follow-up was relatively short. A larger number of cases with long-term follow-up are required to generalize the study results. Second, this study was retrospective and an unknown bias might have affected the study results. Third, clinical data including patient symptoms were not evaluated in this study. Fourth, this study focused on two-dimensional radiographic measurements, which might not be sufficient to evaluate the complex three-dimensional nature of flatfoot deformities and the ankle joint in the coronal plane. In addition, heel valgus deformity was not evaluated radiographically.

In conclusion, flatfoot correction by lateral column and Achilles tendon lengthening caused radiographic changes in the ankle joint of patients without OA. However, the biomechanical effects of this change and the potential long-term benefits on the ankle joint require further investigation; thereafter, the relationship between foot deformities and ankle joint biomechanics can be markedly highlighted.
